# Engineering *Corynebacterium glutamicum* to produce 5-aminolevulinic acid from glucose

**DOI:** 10.1186/s12934-015-0364-8

**Published:** 2015-11-17

**Authors:** Xiaoli Yu, Haiying Jin, Wenjing Liu, Qian Wang, Qingsheng Qi

**Affiliations:** State Key Laboratory of Microbial Technology, Shandong University, Jinan, 250100 People’s Republic of China; National Glycoengineering Research Center, Shandong University, Jinan, 250100 People’s Republic of China

**Keywords:** *Corynebacterium glutamicum*, 5-Aminolevulinic acid, Heme biosynthesis, Metabolic engineering

## Abstract

**Background:**

*Corynebacterium glutamicum* is generally regarded as a safe microorganism and is used to produce many biochemicals, including l-glutamate. 5-Aminolevulinic acid (ALA) is an l-glutamate derived non-protein amino acid, and is widely applied in fields such as medicine and agriculture.

**Results:**

The products of the *gltX*, *hemA*, and *hemL* genes participate in the synthesis of ALA from l-glutamate. Their annotated *C. glutamicum* homologs were shown to be functional using heterologous complementation and overexpression techniques. Coexpression of *hemA* and *hemL* in native host led to the accumulation of ALA, suggesting the potential of *C. glutamicum* to produce ALA for research and commercial purposes. To improve ALA production, we constructed recombinant *C. glutamicum* strains expressing *hemA* and *hemL* derived from different organisms. Transcriptome analysis indicated that the dissolved oxygen level and Fe^2+^ concentration had major effects on ALA synthesis. The downstream pathway of heme biosynthesis was inhibited using small molecules or introducing genetic modifications. Small-scale flask cultures of engineered *C. glutamicum* produced 1.79 g/L of ALA.

**Conclusion:**

Functional characterization of the key enzymes indicated complex regulation of the heme biosynthetic pathway in *C. glutamicum*. Systematic analysis and molecular genetic engineering of *C. glutamicum* may facilitate its development as a system for large-scale synthesis of ALA.

**Electronic supplementary material:**

The online version of this article (doi:10.1186/s12934-015-0364-8) contains supplementary material, which is available to authorized users.

## Background

*Corynebacterium glutamicum*, which occurs naturally in soil, is a Gram-positive, nonpathogenic, biotin auxotroph bacterium that is used for large-scale industrial production of the flavor enhancer l-glutamate and several other amino acids [1]. Recent studies demonstrate the potential of *C. glutamicum* to produce a variety of other commercially interesting compounds such as organic acids, diamines, and biofuels [2]. Because of its importance to industrial biotechnology, *C. glutamicum* serves as a prominent model organism for studying prokaryotic metabolism and its regulation as well as providing a subject for applying the tools and concepts of synthetic biology [3].

**Fig. 1:**
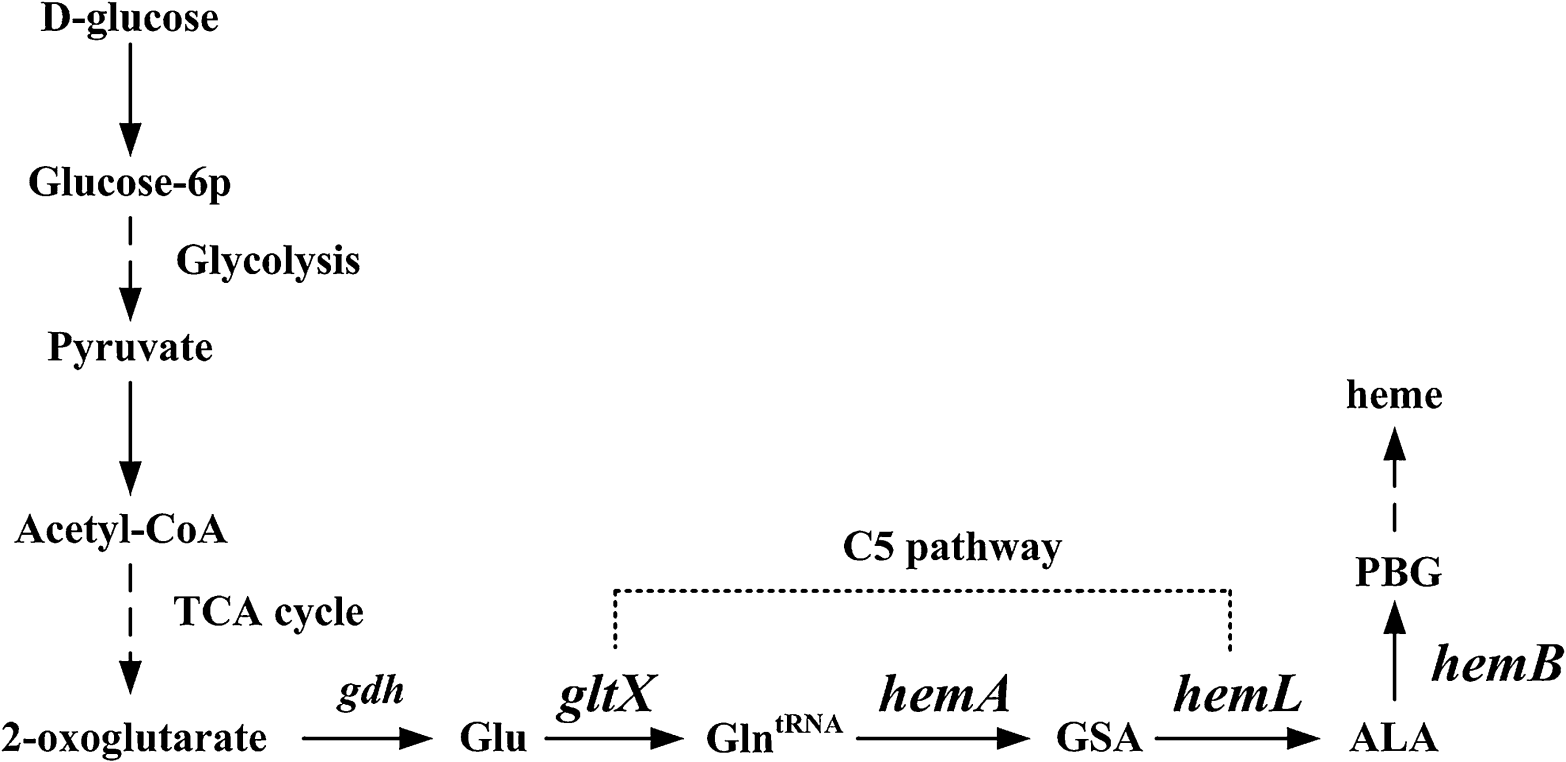
The ALA biosynthesis pathway in *C. glutamicum*. The enzymes encoded by the corresponding genes are: *gdh* glutamate dehydrogenase; *gltX* glutamyl-tRNA synthetase; *hemA* glutamyl-tRNA reductase; *hemL* glutamate-1-semialdehyde aminotransferase; *hemB* 5-aminolevulinic acid dehydratase. *Glu* glutamate; *Gln* ^tRNA^ Glutamyl-tRNA; *GSA* glutamate 1-semialdehyde aminotransferase; *ALA* 5-aminolevulinic acid; *PBG* porphobilinogen

The iron-containing tetrapyrrole heme is a cofactor of the protein components of the electron transport chain that drives aerobic and anaerobic respiration [4]. Certain central metabolic pathways and enzymes require heme for activity, although excess heme is toxic because of its reactive nature [5]. The mechanism of the biosynthesis of heme from its first precursor, 5-aminolevulinic acid (ALA), is highly conserved among organisms. However, the biosynthesis of ALA is regulated at different levels depending on species and may be subject to feedback inhibition by heme [6]. The key genes *gltX*, *hemA*, and *hemL*, which participate in the synthesis of ALA from glutamate, were identified in the genome of *C. glutamicum* using in silico techniques, including sequence alignments and the identification of domains shared with those of their functionally verified counterparts (Fig. 1). However, the sequences of these *C. glutamicum* genes are only 31.53, 25.32, and 47.05 % identical to those of GluRS, HemA, and HemL, respectively, of *Escherichia coli*, and the functions of the former are unknown.

5-Aminolevulinic acid is used for photodynamic therapy (PDT) in gastroenterology, urology, and dermatology and is used as well as a photosensitizer in photodynamic diagnosis [7]. Moreover, the application of low concentrations of ALA for agricultural purposes increases the tolerance of plants to low temperatures and high salt concentrations and is a biodegradable herbicide and insecticide [8]. Because the chemical synthesis of ALA is complicated and generates relatively low yields, the focus of researchers is on using microbial cell factories to synthesize ALA, because such systems are environmentally safe, economical, and sustainable [9]. For example, microbes such as *Rhodobacter sphaeroides* and *E. coli* were engineered to produce ALA [10]. However, most of these studies focused on the condensation reaction of succinyl-CoA and glycine that is catalyzed by ALA synthase (C4 pathway) [11]. The C5 biosynthetic pathway was recently engineered in *E. coli* to achieve yields of 4.13 g/L using batch fermentation [12]. The synthesis of ALA from glucose by the C5 pathway is an important advantage over the C4 pathway. The heme pathway was further optimized, and its regulatory factors such as small RNA *ryhB* were studied as well [13, 14].

Because l-glutamate is a precursor of ALA, and glutamate-producing *C. glutamicum* is generally regarded as safe, we reasoned that it might serve as an ideal host for the production of ALA. For this purpose, we conducted heterologous complementation and overexpression experiments, and demonstrate here, for the first time to our knowledge, the function of the key *C. glutamicum* enzymes involved in heme biosynthesis. Moreover, an engineered *C. glutamicum* strain produced ALA at yields of 1.79 g/L.

## Results

### Functional identification of the key genes involved in ALA biosynthesis

5-Aminolevulinic acid is a direct derivative of the tricarboxylic acid (TCA) cycle in the C5 pathway of *C. glutamicum*, which was predicted by analysis of the KEGG database or in previous studies [15]. However, there is no biochemical evidence, to our knowledge, that demonstrates the role of any component of the pathway. Three *C. glutamicum* genes, *gltX* (NP_600515.1), *hemA* (NP_599664.2), and *hemL* (NP_599684.1) were predicted to encode the enzymes involved in ALA biosynthesis. However, the predicted amino acid sequences of these genes are only 31.53, 25.32, and 47.05 % identical to their respective cognate counterparts in *E. coil*, indicating they need functional verification. *C. glutamicum gltX* gene was predicted to encode a glutamyl-tRNA synthetase (GluRS). Conserved Domain Search showed that the conserved HIGH and KMSKS motifs also existed in the catalytic domain of *C. glutamicum* GluRS (Additional file 1: Figure S1). GluRS from *C. glutamicum* probably belonged to the Class I synthetase that aminoacylates the 3′ hydroxyl group of the cognate tRNA [16]. Glutamyl-tRNA reductase (HemA) was predicted to be encoded by *hemA*. The reactive sulfhydryl group (Cys-50) of *E. coli* HemA requires Mg^2+^ to attack the α-carbonyl group of glutamyl-tRNA [17], and Cys-51 of *C. glutamicum* HemA is presumably similar to Cys-50 of the active site of *E. coli* HemA. Gly-197, Arg-222, Asp-285, and Pro-289 are conserved within the NADPH binding domain and may bind NADPH. The predicated HemL sequence contains a pyridoxal 5′-phosphate binding domain, which may participate in amino acid transfer [18]. These features suggest a functional relationship to their *E. coli* counterparts.

**Fig. 2:**
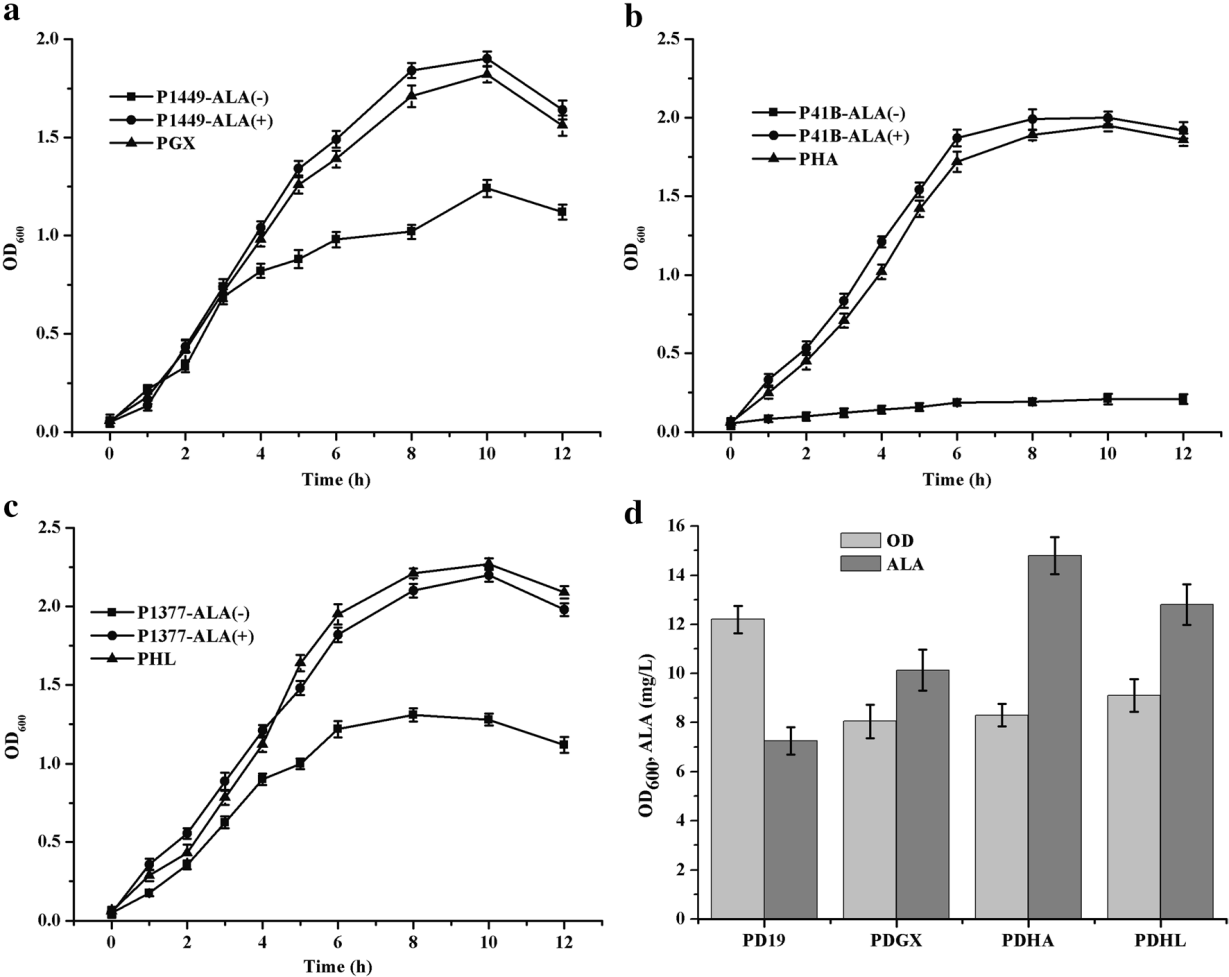
The complementation and ALA accumulation experiments in recombinant *E. coli*. **a** The ALA auxotrophic *E. coli* JP1449 containing pGX. **b** The ALA auxotrophic *E. coli* SASX41B containing pHA. **c** The ALA auxotrophic *E. coli* GE1377containing pHL. **d** The ALA accumulation in recombinant *E. coli* DH5α expressing *gltX*, *hemA* and *hemL* from *C. glutamicum*. Complementation experiments using the ALA auxotrophic strains were grown in LB medium supplemented with 50 μg/mL ALA (ALA+) or without ALA (ALA−). The wild type *E. coli* DH5α was respectively transformed with pUC19, pGX, pHA and pHL, generating the strain PD19, PDGX, PDHA and PDHL. Samples were taken and measured at 24 h with an interval of 4 h. The results were the average of three individual experiments

To verify these assumptions, the three genes from *C. glutamicum* were subcloned into pUC19 (designated pGX, pHA, and pHL, respectively) and tested for complementation of the respective *E. coli* mutants. The *E. coli* mutants JP1449, SASX41B, and GE1377 harboring a defective *gltX*, *hemA*, or *hemL* mutant, respectively, were unable to grow in minimal medium containing glucose but grew on solid medium when transformed with the plasmids encoding the cognate *C. glutamicum* genes. Further, the recombinant strains cultivated in LB medium (Fig. 2a–c) grew to higher optical densities, measured at 600 nm (OD_600_), than the controls, verifying the anaplerotic functions of these three genes. Moreover, adding ALA to cultures of the control strains increased cell growth to the levels of the recombinants, suggesting that ALA is a key intermediate that supports the growth of the mutants. To further characterize these genes, pGX, pHA, and pHL were used to transform wild-type *E. coli* DH5α to generate the *E. coli* strains PDGX, PDHA, and PDHL, which were cultivated in modified minimal medium containing 20 g/L glucose, and analyzed for ALA production. Although OD_600_ values of PDGX, PDHA, and PDHL were lower than that of wild-type *E. coli* DH5α, ALA production by each strain was higher than that of each control (Fig. 2d). These results suggest that these putative *gltX*, *hemA*, and *hemL* genes encode glutamyl-tRNA synthetase, glutamyl-tRNA reductase, and glutamate-1-semialdehyde aminotransferase, respectively.

The shuttle vector pECXK99E was used to overexpress *C. glutamicumgltX*, *hemA*, and *hemL* (PEGX, PEHA, and PEHL, respectively) in their native host. Only PEHA produced increased levels of ALA (Table 1). These data indicate that the conversion of glutamyl-tRNA to glutamate-1-semialdehyde catalyzed by HemA is likely a rate-limiting step. Coexpression of *hemL* and *hemA* significantly improved ALA production, which was not increased by further overexpression of *gltX*, suggesting that a complex mechanism regulates the activity of the heme biosynthetic pathway in *C. glutamicum* (Table 1).

**Table 1:** ALA accumulation in recombinant *C. glutamicum* expressing endogenous *gltX*, *hemA* and *hemL*

*C. glutamicum* strains	Expressed genes	Cell biomass (OD_600_)	Glutamate (g/L)	ALA accumulation (mg/L)	PBG accumulation (mg/L)	Heme accumulation (mg/L)
PECX	–	18.84 ± 0.76	1.9 ± 0.14	25.44 ± 1.56	1.26 ± 0.26	0.12 ± 0.05
PEGX	*gltX*	18.62 ± 0.98	1.85 ± 0.17	20.88 ± 1.34	1.27 ± 0.25	0.14 ± 0.04
PEHA	*hemA*	19.11 ± 1.56	1.95 ± 0.15	56.89 ± 9.21	3.16 ± 0.23	0.56 ± 0.12
PEHL	*hemL*	18.24 ± 0.87	2.05 ± 0.24	24.93 ± 2.53	1.21 ± 0.41	0.13 ± 0.08
CGAL	*hemA*, *hemL*	17.63 ± 0.46	3.55 ± 0.49	79.84 ± 6.56	9.46 ± 0.54	0.82 ± 0.28
PALX	*hemA*, *hemL*, *gltX*	17.86 ± 0.64	2.75 ± 0.31	64.55 ± 3.61	11.12 ± 0.32	1.05 ± 0.23

Samples were taken and measured until 65 h and cultivation was performed at 180 rpm

40 g/L glucose was added initially

Results are the average of three independent experiments

### Engineering *C. glutamicum* to enhance ALA biosynthesis

Overexpression of endogenous *hemA* and *hemL* in *C. glutamicum* improved ALA production as well as that of its precursor, glutamate (Table 1), suggesting the potential use of *C. glutamicum* to produce ALA. To improve ALA production further, we employed genes required for its synthesis from other organisms (Table 2). We generated recombinant *C. glutamicum* strains designated CEAL, SCAL, SEAL, PSEC, and PSEE that coexpressed *hemA* with *hemL* and *gltX* from different organisms (Table 2). All five strains produced ALA, and the yield of SEAL was the highest (425 mg/L). Further, the acetate and lactate concentrations were low and will benefit ALA production by *C. glutamicum* (Table 2). The cells and media turned red after approximately 8 h, indicating the formation of specific intermediates (data not shown). Analysis of downstream metabolites revealed the accumulation of porphobilinogen (PBG) and heme. The relative increases in the concentrations of ALA corresponded to those of PBG and heme, except that strains PSEC and PSEE produced PBG and heme but not ALA (Table 2). The expression of *gltX* in *E. coli* increases the expression of *hemB* (encoding 5-aminolevulinic acid dehydratase, ALAD), which may contribute to the synthesis of more porphyrin derivatives or heme [12]. However, the mechanism in *C. glutamicum* is not clear. Taken together, the data reveal that the activity of the *Salmonella arizona* mutant *hemA*^M^ was active in *C. glutamicum* and that its coexpression with *hemL* greatly increased ALA accumulation. Since ALA, as the precursor of heme biosynthetic pathway, accumulated in the engineered strain, the accumulation of downstream metabolites may also increase. As aforementioned, the heme biosynthetic pathway was complicatedly regulated and was hard to be regulated. In order to improve ALA production, some useful strategies should be explored and applied.

**Table 2:** Engineering the ALA production in *C. glutamicum* using the *gltX*, *hemA* and *hemL* from different sources

*C. glutamicum* strains	Expressed genes	Cell biomass (OD_600_)	ALA (mg/L)	Lactate (g/L)	Acetate (g/L)	PBG (mg/L)	Heme (mg/L)
PECX	–	18.84 ± 0.76	25.44 ± 1.56	7.45 ± 0.46	32.41 ± 1.27	1.26 ± 0.26	0.12 ± 0.05
CEAL	*hemA* (*C. glutamicum*) and *hemL* (*E. coli*)	18.07 ± 0.84	128.13 ± 8.94	4.25 ± 0.19	26.24 ± 2.44	21.34 ± 2.47	1.54 ± 0.28
SCAL	*hemA* ^M^ (*S. arizona*) and *hemL* (*C. glutamicum*)	17.75 ± 0.72	83.47 ± 7.33	0.19 ± 0.07	4.23 ± 0.59	11.26 ± 1.24	0.98 ± 0.18
SEAL	*hemA* ^M^ (*S. arizona*) and *hemL* (*E. coli*)	18.33 ± 0.88	425.11 ± 15.69	0.04 ± 0.008	0.28 ± 0.03	97.87 ± 4.02	3.18 ± 0.54
PSEC	*hemA* ^M^ (*S. arizona*), *hemL* (*E. coli*) and *gltX* (*C. glutamicum*)	16.81 ± 0.74	251.36 ± 10.11	0.17 ± 0.03	1.45 ± 0.12	117.92 ± 2.49	4.15 ± 0.75
PSEE	*hemA* ^M^ (*S. arizona*), *hemL* (*E. coli*) and *gltX* (*E. coli*)	16.45 ± 0.78	276.21 ± 9.26	0.14 ± 0.05	1.12 ± 0.34	121.38 ± 2.53	4.22 ± 0.62

Fermentations were performed at 180 rpm for 144 h, and the initial glucose concentration was 40 g/L

Results are the means ± standard deviations in three individual experiments

### Transcriptional analysis to identify key factors that affect ALA synthesis

SEAL cells and the culture medium were red, indicating the presence of porphyrins and heme, which may affect metabolism. It is hypothesized therefore that specific sets of genes were differentially regulated in the SEAL strain to restore metabolic homeostasis. To optimize culture conditions to increase ALA synthesis, we compared the gene expression levels of the SEAL cells with those of the control strain PECX. Total RNA was isolated when the cultivation medium turned red (after approximately 8 h). Among the nearly 3000 genes analyzed, most were down-regulated and were identified as those involved in glucose transport, glycolysis, respiratory chain, or ATP synthesis (Additional file 1: Table S2). Specifically, the levels of *ldh* and *ackA* mRNAs required for the synthesis of lactate and acetate, respectively, were down-regulated. Consistent with these data, the SEAL strain exhibited reduced glycolytic activity indicated by slower acidification of the medium, which correlated with delayed consumption of glucose and decreased secretion of lactate and acetate (Table 2). Moreover, the down-regulation of the transcription of genes *hemH* for heme synthesis, *sufC* and *sufD* for Fe-S cluster synthesis which are also involved in Fe^2+^ transporters, combined with the up-regulation of the transcription of genes involved in oxidative stress and protein repair may represent strategies employed by cells to avoid toxicity caused by excess concentrations of heme and Fe^2+^. Down-regulated electron flow through the electron transport chain, which is possibly associated with the reduced synthesis of heme and the Fe-S cluster, suggests that a high respiratory rate may adversely affect cell metabolism.

The transcriptional data suggest the likelihood that the potentially toxic mechanism of iron is connected with the high level of dissolved oxygen (DO). To address this question, we determined Fe^2+^ concentrations and DO levels and found that ALA production increased in proportion to the decrease in the concentration of Fe^2+^, and decreased DO levels were associated with increased ALA production (Table 3). When we reduced the rotation of culture flasks to 120 rpm and reduced the concentration of FeSO_4_·7H_2_O to 0.01 mg/L, ALA production increased to 830 mg/L (Table 3), indicating that low DO and Fe^2+^ levels increased ALA production. However, when the culture flasks were rotated at 80 rpm, ALA production decreased, suggesting that the level of DO that enhanced ALA production must be sufficiently high to support cell growth. Therefore, in the following experiments, we used 0.01 mg/L FeSO_4_·7H_2_O and rotated the culture flasks at 120 rpm, which were optimal for producing ALA.

**Table 3:** ALA production in *C. glutamicum* SEAL containing various concentrations of Fe^2+^ at 180, 120 and 80 rpm

The concentration of FeSO_4_·7H_2_O (mg/L)	ALA accumulation (mg/L)		
	180 rpm	120 rpm	80 rpm
10	528.2 ± 17.88	648.2 ± 16.25	364.4 ± 10.32
0.2	573.1 ± 16.34	652.1 ± 17.27	382.3 ± 13.45
0.1	612.4 ± 18.32	702.4 ± 18.49	402.5 ± 14.28
0.05	664.5 ± 19.48	738.3 ± 19.24	446.2 ± 13.89
0.01	678.3 ± 15.27	830.2 ± 21.24	482.6 ± 14.12
0	548.7 ± 14.58	692.7 ± 18.44	418.6 ± 16.02

### Inhibition of ALA downstream pathway using different inhibitors

**Fig. 3:**
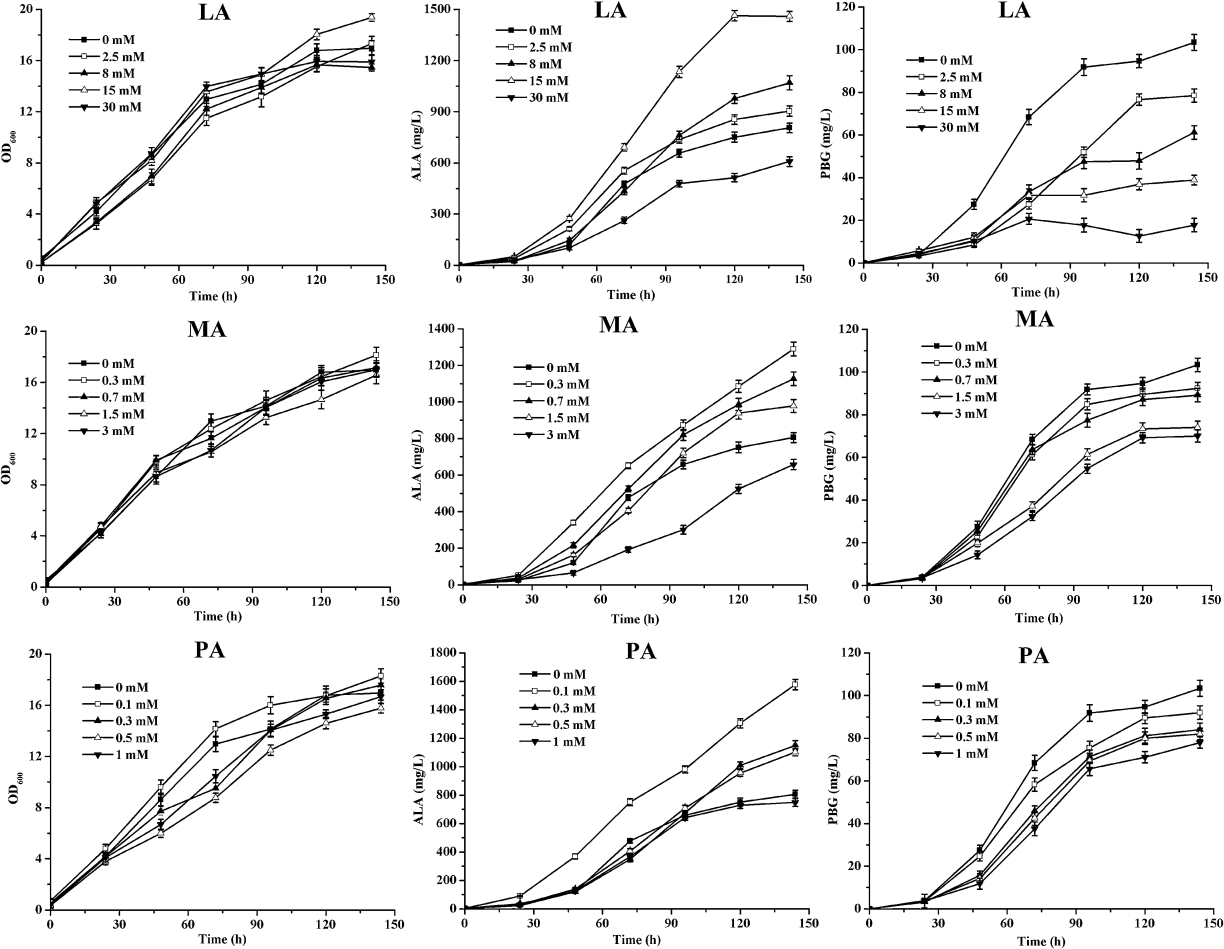
Effects on the growth, ALA and PBG accumulation in *C. glutamicum* SEAL with addition of LA, MA and PA. 40 g/L glucose was added initially as the sole carbon source. The results are the average of three individual experiments

Levulinic acid (LA) is a competitive inhibitor of the downstream metabolic pathway of ALA and is used to enhance ALA production [19]. For the first time to our knowledge, we used the other inhibitors such as maleic acid (MA), phthalic acid (PA), and 2-nitrobenzoic acid (NA) for this purpose. These inhibitors did not influence growth but did affect the accumulation of ALA and PBG (Fig. 3). Addition of LA, MA and PA improved ALA production with the decrease of PBG accumulation. However, NA did not significantly increase the production of ALA (data not shown). ALA production gradually increased when cultures were treated with concentrations of LA from 2.5 to 15 mM. ALA production was significantly decreased in the presence of 30 mM LA. Further, PBG production decreased in the presence of 2.5–30 mM LA. In contrast, low concentrations of MA and PA increased ALA production, which reached 1289 and 1507 mg/L in the presence of 0.3 mM MA and 0.1 mM of PA, respectively. Compared with the control, the PBG accumulation was also decreased, and exhibited different behaviors in compare with the presence of LA. These results suggest that the inhibition of downstream metabolic pathway of ALA affected cell metabolism and that this strategy increases ALA production.

### Molecular genetic modifications that down-regulate ALAD activity

**Fig. 4:**
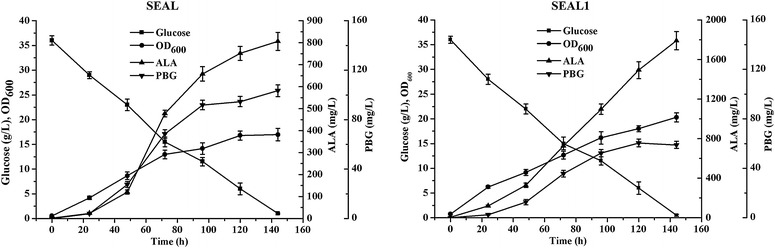
The growth, ALA and PBG accumulation in *C. glutamicum* SEAL and SEAL1. SEAL contains the plasmid pSEAL, and SEAL1 contains the plasmid pSEAL and the ASV tag in the C-terminus of ALAD. 40 g/L glucose was added initially as the sole carbon source. The results are the average of three independent experiments

Because inhibition of the downstream heme biosynthesis increased the production of ALA, and *hemB* (encoding ALAD) is required for viability, we added a degradation tag into the C-terminus of ALAD to increase its rate of degradation. The predicted amino acid sequence of *C. glutamicumhemB* (NP_599678.1) showed 46.9 % identity to its *E. coli* counterpart; however, the function of the protein encoded by *C. glutamicum hemB* is unknown. Corynebacterial SsrA tags vary in their three C-terminal amino acid residues and are designated ASV (AAEKSQRDYAASV**)** and AAV (AAEKSQRDYAAAV**)** [20]. The ASV tag was successfully integrated into the C-terminus of ALAD, but the AAV tag was failed probably due to its strong degradation. The plasmids pSEAL that expressed *hemA*^M^ (*S. arizona*) and *hemL* (*E. coli*) were used to transform *C. glutamicum* that expressed ALAD with the C-terminal ASV tag (SEAL1). Cells that expressed SEAL1 produced increased concentrations of ALA (1.79 g/L), and the concentrations of PBG in the medium were reduced (Fig. 4), suggesting that the predicated *hemB* gene encodes ALAD and that the degradation of ALAD increased ALA production.

## Discussion

In the present study, we determined the functions of key *C. glutamicum* genes predicted to encode enzymes that mediate heme biosynthesis. Previous studies determined that HemA is a major control point for ALA biosynthesis [12]. However, our results indicate that the relative activity of HemA produced by *C. glutamicum* is not high. The HemA activity of *S. typhimurium* is high; however, the enzyme is not stable in the presence of heme [21, 22]. Therefore, the stability of HemA was increased by inserting two Lys residues (AAGAAG) between Thr-2 and Leu-3 at its N-terminus [21]. We show here that expression of HemA^M^ from *S. arizona* with *E. coli* HemL by the recombinant strain designated SEAL produced the highest level of ALA, which indicates that these enzymes synergize, likely because of the formation of a tight complex. However, the glutamate was also detected in the culture medium.

Several lines of evidence indicate that heme plays a major role in the regulation of tetrapyrrole biosynthesis. For example, in the presence of an excess of intracellular heme, the activity of GluRS and the level of HemA are down-regulated and decrease the production of ALA [5]. Moreover, excess heme is toxic and perturbs the central metabolism of *S. aureus* [5]. However, we conclude that the low rates of growth, glucose consumption, and ALA synthesis may be explained by the intracellular accumulation of inhibitory concentrations of porphyrins and heme.

Downstream products such as protoporphyrin IX are potentially toxic in the presence of oxygen, leading to the generation of reactive oxygen species [23]. Further, the addition of iron drives ALA for porphyrins and heme production and causes the cultures to become red [24]. Higher iron concentrations influence cell growth and regulate heme synthesis [12]. Iron is an important cofactor involved in the formation of iron-sulphur (Fe-S) clusters and the activity of heme, which are components of oxygen-binding and regulatory proteins as well as those of the electron transport chain [25]. When the iron concentration is in excess, it may trigger the Fenton reaction and cause oxidative stress [26]. However, the *suf* operon is specifically adapted to synthesize Fe-S clusters under iron-limiting conditions [27], and, according to the transcriptome analysis of SEAL performed here, the synthesis of the Fe-S cluster will likely decrease when iron is in excess. Moreover, when the iron concentration decreased, heme biosynthesis was likely reduced, which may relieve feedback inhibition of the expression of GluRS and HemA to the benefit of ALA synthesis. It has been demonstrated in vertebrate that the homeostatic mechanisms that regulate iron and oxygen interact, and the balance between the contents of protoheme and iron to form active heme is critical [28]; however, their homeostatic concentrations in bacteria are unknown. It is interesting to note that low concentrations of Fe^2+^ and DO reduced the inhibitory effects of porphyrins and heme on ALA synthesis by *C. glutamicum*. Toxicity caused by nonspecific redox cycling occurs between heme and oxygen when excess heme accumulates within the cell membrane [29], and the reduced rates of respiration and heme biosynthesis may mitigate this toxicity. We hypothesize therefore that low levels of DO and Fe^2+^ may compensate for the excess iron available to form Fe-S clusters and to synthesize heme. Such a mechanism may adjust to down-regulate heme synthesis and electron transport under these conditions. The data presented are consistent with this hypothesis, because they indicate that reducing the levels of stressors and heme represents an efficient strategy to increase ALA production.

A *hemB* mutant of *E. coli* does not produce higher levels of ALA compared with wild-type [30], while d-glucose and d-xylose, which were used to inhibit the expression of ALAD, are usually metabolized [11]. Therefore, only LA is typically used to increase ALA production by *R. sphaeroides* [19, 31, 32], *Chlorella vulgaris* [33], and *E. coli* [34]. For example, 15 mmol/L LA inhibits ALAD activity by 60 % at pH 6.5 [19]. Studies of compounds with different inhibitory activities reveal that MA and PA increased ALA production and decreased the production of PBG [35]. These inhibitors interact at distinct steps of the reaction mechanism [36], and different classes of inhibitors exhibit significant differences in influencing the kinetics of the enzyme reactions that produce ALA [35]. PA may serve as a more desirable ALAD inhibitor, because PA is relatively inexpensive and is a highly efficient inhibitor.

In *E. coli*, protein degradation occurs, in part, through the tmRNA system. The C-terminal fusion of SsrA guides proteins to endogenous ClpXP, ClpAP, or FtsH proteases for rapid degradation. Variants of the *E. coli* SsrA tag are used to modify the degradation of proteins [37]. Moreover, *ssrA* is highly conserved, and a homologue is annotated in the *C. glutamicum* genome [38]. The SsrA tag is used to monitor dynamic gene expression patterns in *C. glutamicum* [20]. Here, the addition of the SsrA tag significantly decreased the production of PBG and increased ALA production in the absence of other inhibitors, indicating that ALAD is a key regulatory point in the heme biosynthetic pathway and that genetic modification of ALAD will be useful for improving ALA production.

## Conclusion

In summary, we identified several key genes involved in heme biosynthesis and demonstrated their function in complementation and overexpression experiments. Recombinant *C. glutamicum* strains that produce ALA were constructed by expressing *hemA* and *hemL* from different organisms. Using a strategy that included transcriptome analysis and genomic modifications, we engineered *C. glutamicum* (strain SEAL1) to produce 1.79 g/L of ALA in a small-scale flask culture. ALA synthesis will likely be improved by further optimizing culture conditions using a rigorously controlled fermenter.

## Methods

### Strains, primers and plasmids

All strains, plasmids, and oligonucleotides used in this study are summarized in the additional files (see Additional file 1: Tables S3 and Table S4). The *gltX*, *hemA*, and *hemL* genes were amplified from the wild-type *C. glutamicum* ATCC 13032 genome using primers gltx-F, gltx-R and hema-F, hema-R, and heml-F, and heml-R, respectively. The three amplicons were ligated to pUC19, which was digested with *Sal*I (Fermentas, China) according to the method of Gibson assembly that included T5 exonuclease (Epicentre, USA), Phusion DNA polymerase (New England Biolabs, USA), and *Taq* DNA ligase (New England Biolabs, USA) [39]. Moreover, *gltX*, *hemA*, and *hemL* were cloned from the genome of *C. glutamicum* ATCC 13032 using the primers cggltx-F, cggltx-R and cghema-F, cgdhema-R, and cgdheml-F, cgheml-R. To enhance the stability of HemA, *hemA* was cloned from the *S. arizona* genome, which encodes the same predicted HemA amino acid sequence of *S. typhimurium*. For this purpose, the primers with mutations (sthemA^M^-F and sthemA^M^-R) were used to insert two codons (AAGAAG) encoding Lys between Thr-2 and Leu-3 at the N-terminus [22]. One *hemL* gene was amplified using the primers echeml-F and echeml-R designed according to the sequence of the wild-type *E. coli* MG1655 genome, and *hemL* was cloned using primers cgheml-F and cgheml-R designed according to the sequence of the *C. glutamicum* ATCC 13032 genome. These amplicons contained 30-bp overlaps and were ligated individually or together into pECXK99E that was cleaved with *Kpn*I (Fermentas, China) using the Gibson assembly method. The *gltX* genes were cloned from the genomes of *E. coli* MG1655 and *C. glutamicum* ATCC 13032 using the primers mgltx-F, mgltx-R and cgltx-F, cgltx-R and then ligated into pSEAL digested with *Pst*I using Gibson assembly. *E. coli* DH5α served as the host for molecular cloning and construction of plasmids. The inducible suicide vector pKJL, which was constructed from pK18mobsacB, was used to add ASV and AAV tags to the C-terminus of ALAD encoded by the *C. glutamicum* ATCC 13032 genome [40]. The sequences of the plasmids were verified by the BioSune Company (Shanghai, China). Transformation of ALA auxotrophs and *E. coli* DH5α was achieved using the CaCl_2_ procedure, whereas *C. glutamicum* was electrophoretically transformed [41]. *C. glutamicum* strain ATCC 13032 was used as parental strain for ALA production.

### Media and culture conditions

LB medium (10 g/L tryptone, 5 g/L yeast extract, and 10 g/L NaCl, pH 7.2) and BHIS medium (2.5 g/L beef extract, 5 g/L tryptone, 5 g/L NaCl, 18.5 g/L brain heart infusion, and 91 g/L sorbitol) were for molecular genetic procedures. Kanamycin (50 μg/mL) and ampicillin (100 μg/mL) were added as selective agents as required. *E. coli* was cultured using modified minimal medium [12] (16 g/L (NH_4_)_2_SO_4_, 3 g/L KH_2_PO_4_, 16 g/L Na_2_HPO_4_·12H_2_O, 1 g/L MgSO_4_·7H_2_O, 0.01 g/L MnSO_4_·7H_2_O, 2 g/L yeast extract, and 20 g/L glucose. *C. glutamicum* was cultured using a modified minimal medium (CGXII) containing glucose as the sole carbon source. CGXII contains (per liter) 20 g of (NH_4_)_2_SO_4_, 5 g of urea, 1 g of KH_2_PO_4_, 1 g of K_2_HPO_4_, 0.25 g of MgSO_4_·7H_2_O, 42 g of MOPS (3-morpholinopropanesulfonic acid), 10 mg of CaCl_2_, 10 mg of FeSO_4_·7H_2_O, 10 mg of MnSO_4_·H_2_O, 1 mg of ZnSO4·7H_2_O, 0.2 mg CuSO_4_, 0.02 mg NiCl_2_·6H_2_O, 0.02 g citrate sodium, and pH 7.0. To induce the expression of plasmid genes, isopropyl-*β*-d-thiogalactopyranoside was added to the cultures at a final concentration of 0.25 mM.

ALA was produced at 30 °C in 300 mL using ordinary flasks containing 50 mL modified CGXII. Starter cultures contained 5 ml LB with glucose were routinely inoculated with a single colony from a freshly streaked agar plate, the culture flasks were rotated at 180 rpm for 14 h at 30 °C, and the cells were harvested, and washed with sterile 0.9 % NaCl and CGXII containing 5.0 μg/L biotin. The resuspended cells were used to inoculate a second culture containing 5.0 μg/L biotin, and the initial OD_600_ was adjusted to approximately 0.5. After approximately 12 h incubation at 30 °C at 170 rpm, the cells in the exponential growth phase were harvested and washed sequentially with sterile 0.9 % NaCl solution and CGXII containing 1.0 μg/L biotin and inoculated into the main culture that was adjusted to approximately 0.8 OD_600_. The initial pH was adjusted to 7.0 (optimum), then maintained at approximately pH 6.5 using 4 M NaOH. The inhibitors LA, MA, PA, and NA were added at approximately 8 h.

### Substrate, product, and RNA-Seq analysis

For quantification of extracellular glucose, glutamate, and ALA, aliquots of the culture were withdrawn and the cells were removed by centrifugation (12,000×*g*, 10 min). The OD_600_ was determined using a spectrophotometer (Shimadzu, Japan), and the supernatant was analyzed for glucose and glutamate using a SBA-40C biosensor (developed by Biology Institute of Shandong Academy of Sciences) equipped with glucose and glutamate oxidase immobilized on membranes. To determine the concentrations of organic acids, high-performance liquid chromatography (HPLC) was used (Shimadzu). The supernatant was filtered through a 0.22-mm syringe filter. The HPLC system was equipped with a HPX-87H column (300 mm × 7.8 mm, Bio-Rad, USA) and a differential refractive index (RI) detector (Shimadzu RID-10A). The mobile phase (0.5 mM H_2_SO_4_) was delivered at 0.6 mL/min at 65 °C. To measure ALA and PBG concentrations, we used modified Ehrlich’s reagent and measured absorbance at 554 nm [42]. For heme measurements, a fluorescence assay was used [43]. RNA-Seq was performed according to the Illumina mRNA Sequencing Sample Preparation Guide (Illumina), and sequenced using an Illumina HiSeq sequencer (Illumina) at BGI Tech Company (Shenzhen, China). The raw sequencing data were analyzed using the software included with the system (Illumina).

## Additional file

10.1186/s12934-015-0364-8 Figure S1. Multiple-sequence alignment of GluRS (A), HemA (B), and HemL (C) from *E. coli* and *C. glutamicum*. Table S1. Strains and plasmids used in this study. Table S2. Primers used in this study. Table S3. Down-regulated genes (-) and up-regulated genes (+) were identified using comparative transcriptome analysis of the *C. glutamicum* strains SEAL and PECX.
