# Supplementary material for: Engineering Corynebacterium glutamicum to produce 5-aminolevulinic acid from glucose
Source: Microb Cell Fact. 2015 Nov 17;14:183. doi: 10.1186/s12934-015-0364-8 (PMC4650169; doi:10.1186/s12934-015-0364-8)
Supplement: Supplementary file 1 — 10.1186/s12934-015-0364-8 Figure S1. Multiple-sequence alignment of GluRS (A), HemA (B), and HemL (C) from E. coli and C. glutamicum. Table S1. Strains and plasmids used in this study. Table S2. Primers used in this study. Table S3. Down-regulated genes (-) and up-regulated genes (+) were identified using comparative transcriptome analysis of the C. glutamicum strains SEAL and PECX. [file 12934_2015_364_MOESM1_ESM.doc]

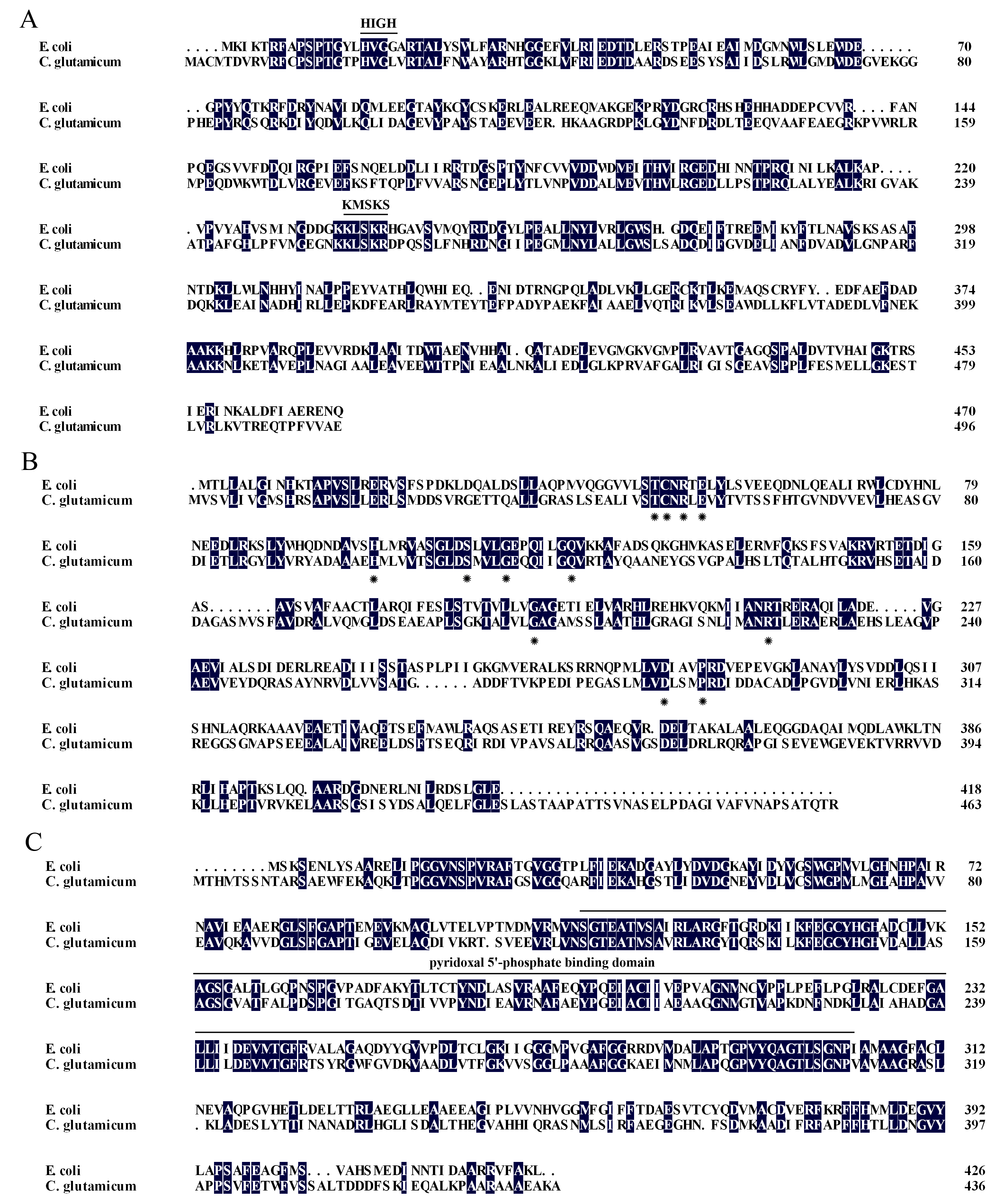


**Figure S1** Multiple-sequence alignment of GluRS (A), HemA (B) and HemL (C) from *E. coli* and *C. glutamicum*. Identical amino acids are shaded in black. The conserved motifs, domains and residues are represented by lines and asterisks.

**Table S1 Strains and plasmids used in this study.**

| Strain, plasmid | Relevant properties | Source or reference |
| --- | --- | --- |
| Strains  *E. coli* DH5α  *E.coli* MG1655  *S. arizona*  *C. glutamicum* ATCC 13032  *C. glutamicum* HBAS  *E. coli* JP1449  *E. coli* SASX41B  *E. coli* GE1377  *E. coli* P1449  *E. coli* PGX  *E. coli* P41B  *E. coli* PHA  *E. coli* P1377  *E. coli* PHL  *E. coli* PD19  *E. coli* PDGX  *E. coli* PDHA  *E. coli* PDHL  *C. glutamicum* PECX  *C. glutamicum* PEGX  *C. glutamicum* PEHA  *C. glutamicum* PEHL  *C. glutamicum* CGAL  *C. glutamicum* PALX  *C. glutamicum* CEAL  *C. glutamicum* SGAL  *C. glutamicum* SEAL  *C. glutamicum* PSEC  *C. glutamicum* PSEE  *C. glutamicum* SEAL1  Plasmids  pUC19  pECXK99E  pK-JL  pGX  pHA  pHL  pEGX  pEHA  pEHL  pCGAL  pCEAL  pSCAL  pSEAL  pPALX  pPSEC  pPSEE | *F^-^, endA1, hsdR17 (rK^-^, mK^+^), supE44, thi-l, λ^-^, recA1, gyrA96,* Δ*lacU169* (*Ø 80lacZ* Δ*M15*)  Wild type  Wild type  Wild type  *C. glutamicum* ATCC 13032 *hemB*^ASV^  *thr-1*, *leuB6* (Am), *lacZ4*, *glnV44* (AS), *λ^-^*, *gltX351* (ts), *rpsL8*, *thiE1* *hemA41*, *relA1*, *spoT1*, *metB1*, *rrnB-2*, *mcrB1*, *creC510*  *thr-1*, *leuB6* (Am), *panD2*, *hemL207*, *lacZ4*, *glnV44* (AS), *λ^-^*, *rfbC1*, *rpsL8*, *thiE1*  *E. coli* JP1449 harboring pUC19  *E. coli* JP1449 harboring pGX  *E. coli* SASX41B harboring pUC19  *E. coli* SASX41B harboring pHA  *E. coli* GE1377 harboring pUC19  *E. coli* GE1377 harboring pHL  *E. coli* DH5α harboring pUC19  *E. coli* DH5α harboring pGX  *E. coli* DH5α harboring pHA  *E. coli* DH5α harboring pHL  *C. glutamicum* ATCC 13032 harboring pECXK99E  *C. glutamicum* ATCC 13032 harboring pEGX  *C. glutamicum* ATCC 13032 harboring pEHA  *C. glutamicum* ATCC 13032 harboring pEHL  *C. glutamicum* ATCC 13032 harboring pCGAL  *C. glutamicum* ATCC 13032 harboring pPALX  *C. glutamicum* ATCC 13032 harboring pCEAL  *C. glutamicum* ATCC 13032 harboring pSCAL  *C. glutamicum* ATCC 13032 harboring pSEAL  *C. glutamicum* ATCC 13032 harboring pPSEC  *C. glutamicum* ATCC 13032 harboring pPSEE  *C. glutamicum* HBAS harboring pSEAL  Cloning vector, Amp^R^ *lac* *POZ*  *C. glutamicum*-*E. coli* shuttle expression vector, Kan^R^  pK18mobsacB derivative, sacB under the control of tac-M promoter, Kan^R^,  pUC19 containing *gltX* (*C. glutamicum*)  pUC19 containing *hemA* (*C. glutamicum*)  pUC19 containing *hemL* (*C. glutamicum*)  pECXK99E containing *gltX* (*C. glutamicum*)  pECXK99E containing *hemA* (*C. glutamicum*)  pECXK99E containing *hemL* (*C. glutamicum*)  pECXK99E containing *hemA* (*C. glutamicum*) and *hemL* (*C. glutamicum*)  pECXK99E containing *hemA* (*C. glutamicum*) and *hemL* (*E. coli*)  pECXK99E containing *hemA*^M^ (*S. arizona*) and *hemL* (*C. glutamicum*)  pECXK99E containing *hemA*^M^ (*S. arizona*) and *hemL* (*E. coli*)  pECXK99E containing *hemA* (*C. glutamicum*), *hemL* (*C. glutamicum*) and *gltX* (*C. glutamicum*)  pECXK99E containing *hemA*^M^ (*S. arizona*), *hemL* (*E. coli*) and *gltX* (*C. glutamicum*)  pECXK99E containing *hemA*^M^ (*S. arizona*), *hemL* (*E. coli*) and *gltX* (*E. coli*) | Lab stock  Lab stock  Lab stock  Lab stock  This study  [16]  [44]  [45]  This study  This study  This study  This study  This study  This study  This study  This study  This study  This study  This study  This study  This study  This study  This study  This study  This study  This study  This study  This study  This study  This study  Lab stock  Lab stock  [40]  This study  This study  This study  This study  This study  This study  This study  This study  This study  This study  This study  This study  This study |

**Table S2 Primers used in this study.**

| Primers | Sequence |
| --- | --- |
| gltx-F  gltx-R  hema-F  hema-R  heml-F  heml-R  cggltx-F  cggltx-R  cgdhema-R  cgdheml-F  cghema-F  cghema-R  cgheml-F  cgheml-R  cgechema-R  echeml-F  echeml-R  sthema-F  sthema-R  mgltx-F  mgltx-R  cgltx-F  cgltx-R  Hemb-a-F  Hemb-a-asv  Hemb-a-aav  Hemb-b-asv  Hemb-b-aav  Hemb-b-R | TTACGCCAAGCTTGCATGCCTGCAGGTCGA**AAGGAGGATATACAT**ATGGCCTGCATGACTGAT  GTAAAACGACGGCCAGTGAATTCGAGCTCCTTACTCAGCGACCACGAATG  TTACGCCAAGCTTGCATGCCTGCAGGTCGA**AAGGAGGATATACAT**ATGGTGAGTGTACTCAT  GTAAAACGACGGCCAGTGAATTCGAGCTCCGTTACTCCCTCGTTTGT  TTACGCCAAGCTTGCATGCCTGCAGGTCGA**AAGGAGGATATACAT**ATGACGCACATGACATCG  GTAAAACGACGGCCAGTGAATTCGAGCTCCATCATGATGCCTTCGCTTC  GCTCGGTACCCGGGGATCCTCTAGAGTCGA**AAGGAGGATATACAT**ATGGCCTGCATGACTGA  AACAGCCAAGCTTGCATGCCTGCAGGTCGATTACTCAGCGACCACGAATG  AACAGCCAAGCTTGCATGCCTGCAGGTCGAGTTACTCCCTCGTTTGT  GCTCGGTACCCGGGGATCCTCTAGAGTCGA**AAGGAGGATATACAT**ATGACGCACATGACATC  GCTCGGTACCCGGGGATCCTCTAGAGTCGA**AAGGAGGATATACAT**ATGGTGAGTGTACTCAT  TGTCATGTGCGTCATATGTATATCCTCCTTGTTACTCCCTCGTTTGT  **AAGGAGGATATACAT**ATGACGCACATGACATCG  AACAGCCAAGCTTGCATGCCTGCAGGTCGAATCATGATGCCTTCGCTTC  TTCAGACTTACTCATATGTATATCCTCCTTGTTACTCCCTCGTTTGT  **AAGGAGGATATACAT**ATGAGTAAGTCTG  AACAGCCAAGCTTGCATGCCTGCAGGTCGATCACAACTTCGCAAACACC  GCTCGGTACCCGGGGATCCTCTAGAGTCGA**AAGGAGGATATACAT**atgaccaagaagC  TGTCATGTGCGTCATATGTATATCCTCCTTCTACTCCAGCCCGAGGCT  tttattgctgaacgcgaaaatcagcagtaaCTGCA**AAGGAGGATATACAT**atgaaaatcaaaactcgcttc  tcCgccaaaacagccaagcttgcatgcTCCCTGCAttactgctgattttcgcgttcagccg  gagcagaccccattcgtggtcgctgagtaaCTGCA**AAGGAGGATATACAT**atggcctgcatgactgatgttc  tcCgccaaaacagccaagcttgcatgcTCCCTGCAttactcagcgaccacgaatggggtctg  TACGAATTCGAGCTCGGTACCCGGGGATCCCCATGAGGCTTTAGATGCTGG  TTA*AACTGATGCAGCGTAATCACGTTGGCTCTTTTCTGCTGC*AGCGTTTCGCAGTGCGCG  TTA*AACAGCTGCTGCGTAATCACGTTGGCTCTTTTCTGCTGC*AGCGTTTCGCAGTGCGCG  AGCCAACGTGATTACGCTGCATCAGTTTAACAAGAACTAACTGATTAGTAGGAC  AGCCAACGTGATTACGCAGCAGCTGTTTAACAAGAACTAACTGATTAGTAGGAC  tgcatgcctgcaggtcgactctagaggatcCAAACAGCATCCAGAACACCAC |

The base underlined means the homology, and the base in bold means the ribosome binding sites added artificially. The SsrA-tag sequence was indicated in italic.

**Table S3** **Down-regulated genes (-) and up-regulated genes (+) were identified by comparative transcriptome analysis of SEAL and PECX.**

| Cg number | Fold change | Description | Fundction |
| --- | --- | --- | --- |
| cg1537  cg2119  cg1791  cg1790  cg1111  cg3219  cg3047  cg0446  cg2891  cg1853  cg0129  cg2780  cg1301  cg1300  cg0551  cg0531  cg1362  cg1365  cg1366  cg1367  cg1368  cg1369  cg1734  cg1762  cg1763  cg3237  cg1248  cg3099  cg3100  cg3098 | - 1.24  -1.03  -1.55  -1.36  -1.19  -1.03  -1.05  -1.16  -1.55  -1.08  -1.65  -1.4  -0.77  -1.44  -0.95  -0.8  -1.88  -0.89  -0.9  -0.63  -1.04  -0.95  -0.99  -1.23  -1.15  +1.79  +1.17  +2.35  +1.72  +1.68 | phosphotransferase system IIABC component, glucose/maltose/N-acetylglucosamine-specific *ptsG*  putative fructose-1-phosphate kinase *pfkB*  glyceraldehyde-3-phosphate dehydrogenase *gapA*  phosphoglycerate kinase *pgk*  phosphopyruvate hydratase *eno*  lactate dehydrogenase *ldh*  acetate kinase *ackA*  succinate dehydrogenase, flavoprotein subunit *sdhA*  pyruvate dehydrogenase pyruvate:quinone oxidoreductase,  (“pyruvate oxidase“) *pqo*  glycerol-3-phosphate dehydrogenase *glpD*  prolline dehydrogenase *putA*  cytochrome aa3 oxidase, subunit I *ctaD*  cytochrome bd oxidase, subunit I *cydA*  cytochrome bd oxidase, subunit II *cydB*  O-succinylbenzoate synthase *menC*  1,4-dihydroxy-2-naphthoate octaprenyltransferase *menA*  ATP synthase subunit A *atpB*  ATP synthase subunit D *atpH*  ATP synthase subunit A *atpA*  ATP synthase subunit C *atpG*  ATP synthase subunit B *atpD*  ATP synthase subunit epsilon *atpC*  ferrochelatase *hemH*  Fe-S cluster assembly ATP-binding protein *sufC*  Fe-S cluster assembly protein *sufD*  manganese superoxide dismutase *Mnsod*  GTPase involved in stress response  molecular chaperone GrpE (heat shock protein)  molecular chaperone DnaK  chaperone with DnaK, heat shock protein (DanJ protein) | Sugar transport  Glycolysis  Lactate synthesis  Acetate synthesis  Respiratory chain components  F_1_F_0_-ATP synthase  heme synthesis  Fe-S cluster synthesis  The response to oxidative stress  Protein repair |
